# Exploring the burden of irritable bowel syndrome among university students in Saudi Arabia: A study on prevalence, psychological associations, and well-being

**DOI:** 10.1097/MD.0000000000038099

**Published:** 2024-05-10

**Authors:** Ayoub Ali Alshaikh, Sultan Mana Alamri, Fatima Riaz, Syed Esam Mahmood, Mohammed Abdullah M. Shlwan, Faisal Naser A Almuidh, Omar Aodah S. Alshahrani, Mohammed Alhussain M. Asiri, Abdulrahman Saeed H. Almuaddi, Nawaf Yahya Y. Al Qasim, Mohammed Abdullah M. AlJebreel, Ramy Mohamed Ghazy

**Affiliations:** a Family and Community Medicine Department, King Khalid University, Abha, Saudi Arabia; b King Faisal Medical City, South Region, Saudi Arabia; c Colleague of Medicine, King Khalid University, Abha, Saudi Arabia; d Tropical Health Department, High Institute of Public Health, Alexandria University, Alexandria, Egypt.

**Keywords:** anxiety, depression, irritable bowel syndrome, Rome IV, stress, University students, well-being

## Abstract

Patients with irritable bowel syndrome (IBS) experience not only a detrimental impact on their physical health but also a significant influence on their psychological well-being. This study aimed to assess the prevalence of IBS among university students, investigating the sociodemographic and lifestyle factors influencing its onset. Furthermore, it explored the potential impact of psychological factors such as depression, anxiety, and overall well-being on the prevalence of IBS. A cross-sectional analytical study was conducted at Saudi Arabian universities in November and December 2023. To collect data, an anonymous, validated, predesigned questionnaire was used. The diagnosis of IBS was carried out using the validated Arabic version of the Rome IV questionnaire. We used the Arabic version of the World Health Organization Well-being Index to assess the participants’ well-being. We used the Arabic version of the Hospital Anxiety and Depression Scale (HADS) to identify people who show clinically significant symptoms of anxiety and depression. Our study included a total of 379 university students, 46.7% were female 86.0% of participants resided in urban areas, and 7.2% had been previously diagnosed with IBS. The prevalence of IBS among participants was found to be 31.9%. We observed a significant association between marital status and IBS (*χ*^2^ = 3.95, *P* = .047). Furthermore, the highest prevalence of IBS was observed among students majoring in literary and scientific disciplines (*χ*^2^ = 0.952, *P* = .049). Individuals with IBS demonstrate a significantly higher prevalence of anxiety (71.90% vs 41.09%, *P* < .001) and depression (64.46% vs 42.64%, *P* < .001) compared to those without IBS. Furthermore, people with IBS had a slightly higher prevalence of poor well-being (38.84%) compared to those without IBS (33.72%), but this association was not statistically significant. In multivariate analysis, having a family history of IBS [OR = 1.75 (95% CI, 1.06–2.87), *P* = .029] having borderline anxiety [OR = 7.58, 95% CI (2.12–27.06), *P* = .012] and anxiety [OR = 16.07, 95% CI (4.57–56.52), *P* < .001], and depression [OR = 2.97, 95% CI (1.13–7.83), *P* = .010] were the main significant predictors of IBS among university students. The high prevalence of IBS among university students was associated with a family history of IBS as well as depression and anxiety. Increasing awareness, multidisciplinary support, and access to mental health services is required to ensure university students’ well-being.

## 1. Introduction

Irritable bowel syndrome (IBS) is one of the most prevalent and potentially incapacitating gastrointestinal diseases. It is characterized by abdominal pain, bloating, and changes in bowel habits, however, it lacks any underlying pathology.^[1]^ Historically, the diagnosis of IBS relied on the exclusion of other clinical and practical factors. However, in recent years, Rome IV criteria have emerged as the industry standard for diagnosing IBS in both research and clinical practice. To meet these criteria, the patient should have experienced recurrent abdominal pain in the last 3 months, which occurs on average at least 1 day a week. This pain should be accompanied by 2 or more of the following criteria: it is associated with defecation, linked to a change in stool frequency, or associated with a change in stool appearance. Additionally, these symptoms should have persisted for at least 3 months prior to diagnosis.^[2]^ The prevalence of IBS varies from 5.7% to 34.0% worldwide, with a wide variation according to the diagnostic tool used.^[3]^ IBS places a significant economic burden on the healthcare system, leading to high resource utilization and generating direct medical costs ranging from approximately $1.5 billion to $10 billion per year.^[4]^ Furthermore, many individuals underwent unnecessary procedures, including appendicectomies and hysterectomies as a result of challenging diagnoses in some uncommon circumstances.^[5]^

In Arab countries, IBS remains 1 of the least explored areas. A comprehensive meta-analysis of the worldwide prevalence of IBS has highlighted the lack of research specifically from any Arab country.^[6]^ Among the limited studies conducted in the Arab region, a study in Suez Governorate, Egypt, revealed a high prevalence rate of IBS at 34.2% among the surveyed population.^[7]^ A study conducted at Ain Shams University, Egypt revealed that 31.7% of medical students suffer from IBS, with higher prevalence rates observed among females and individuals with a positive family history of the condition. The study also identified a significant association between IBS and anxiety but not depression.^[8]^ A study conducted in Saudi Arabia reported a lower prevalence rate of IBS at 11.4%.^[9]^

It is important to note that people with IBS experience significant impacts on their well-being across physical, psychological, and financial domains. Many patients have reported difficulties with concentration, reduced energy levels, and diminished self-esteem.^[10]^ Furthermore, IBS has been associated with a 21% decrease in work productivity, which is equivalent to working less than 4 full days in a standard 5-day workweek.^[11]^ Additionally, many patients experience social embarrassment as a result of irregular bowel habits.^[12]^

Although it is the cause of half of all cases referred to gastroenterologists, IBS has eluded a definitive cause. Nevertheless, some studies suggest that factors like psychological elements, dietary habits, and the level of exercise are associated with the onset and progression of IBS.^[13]^ About 33% of patients with IBS reported positive family history, suggesting that genetics may play a role in the etiology of the condition.^[1]^ Moreover, individual characteristics such as age and gender may influence the development of IBS. Reports indicate that women are more susceptible than men, and individuals in their late teens and early 20s are particularly at risk.^[14]^

University students, particularly those in the medical field, are often predisposed to anxiety and depression. This is due to the demands of long study hours, the high workload, and mental exhaustion caused by multiple assessments. The constant stress they face can have a significant impact on their mental health, potentially serving as an underlying factor for conditions such as IBS.^[15,16]^

This study hypothesized a high prevalence of IBS among university students, particularly medical students, and proposed a significant association between IBS and factors including sociodemographic, lifestyle, well-being, anxiety and depression. This study aimed to determine the prevalence of IBS among university students, using Rome IV criteria. Additionally, it aimed to uncover associated factors, such as sociodemographic characteristics and lifestyle habits, that could influence the onset of IBS. Furthermore, the study examined whether the field of study (health vs non-health), psychological factors such as depression, anxiety, and overall well-being could predict the prevalence of IBS.

## 2. Subject and method

### 2.1. Study setting

We conducted an anonymous cross-sectional online study across 3 universities in Saudi Arabia. King Khalid University in Abha, Bisha University; and King Saud Ibn Abdel Aziz University in Riyadh. Data collection was carried out between November 1 and December 31, 2023.

### 2.2. Sample size

The sample size was calculated using Epi-info software, assuming the hypothesized prevalence of IBS in the population = 31.8% based on a previous study and 95% confidence level. The minimum calculated sample size to achieve study objectives was 334.^[16]^ The sample size was increased to 380 students to compensate for a nonresponse rate of 10%.

### 2.3. Study population and sampling methods

This study’s target population included all university students enrolled at King Khalid University, Bisha University, and King Saud Ibn Abdel Aziz University, encompassing all grades and ages 18 to 25 years. We excluded participants with known organic gastrointestinal disorders, as well as those who showed alarming symptoms such as significant weight loss or bloody stools. Additionally, individuals who had used antibiotics within the past 3 months or received a diagnosis of enteric bacterial or parasitic infections within the last 3 months were also excluded. The number of students recruited from each university was proportional to the total number of students who attended that university. This strategy aimed to maintain representativeness across institutions and ensure a fair distribution of participants in our study. The sample size for this study was divided evenly, with nearly half of the participants drawn from medical colleagues and the remaining half from nonmedical colleges.

### 2.4. Study outcomes

This study assessed the prevalence and predictors of IBS among Saudi university students including socioeconomic factors, field of study (health vs non-health), psychological problems (depression and anxiety), and well-being.

### 2.5. Tools of data collection

We used a self-administered questionnaire to gather baseline data, which included sociodemographic and academic information, family history of IBS, previous physician-based diagnoses of IBS, presence of other chronic medical conditions, as well as daily life habits such as sleeping hours, smoking status, and regular exercise. The latter was defined as engaging in any type of sport for a minimum of 30 minutes 3 times per week, while smoking status was also captured. We categorized participants based on their monthly income, ranging from <5000 Saudi Rial to more than 20,000 Saudi Rial.

Diagnosis of IBS was carried out using the validated Arabic version of the Rome IV questionnaire, a widely accepted and standardized tool for diagnosing functional gastrointestinal disorders. Diagnostic criteria for IBS include recurrent abdominal pain that occurs at least 1 day a week in the last 3 months. This pain should be associated with 2 or more of the following criteria: an association with the act of defecation, a change in the frequency of stool, and a change in the form or appearance of stool.^[17]^

To identify individuals exhibiting clinically significant symptoms of anxiety and depression, the study utilized the validated Arabic version of the Hospital Anxiety and Depression Scale (HADS).^[18]^ HADS consists of 14 questions, evenly divided into 7 for anxiety and 7 for depression. Participants responded on a 4-point Likert scale ranging from 0 (not present) to 3 (considerable). Scores for each subscale, anxiety, and depression, were separately calculated, and individuals were categorized based on scores as follows: 0 to 7 (normal), 8 to 10 (borderline), and over 11 for each subscale indicating clinically significant cases.

The well-being of the study’s participants was assessed using the validated Arabic version of the WHO-5 Well-being Index.^[19]^ Comprising 5 questions, the respondents provided answers on a scale of 5 options, ranging from 0 (at no time) to 5 (all of the time). The raw score, calculated by adding the responses, ranges from 0 to 25, with 0 indicating the poorest and 25 reflecting the best possible quality of life. Transforming the raw score into a percentage score (0–100) involves multiplying it by 4. A percentage score of 0 indicates the worst possible, while a score of 100 signifies the best possible quality of life. A score below 50 suggests poor emotional well-being.^[20]^

Before actual data collection, a pilot study was carried out involving 10 participants to assess the clarity and feasibility of the questionnaires. Based on received feedback, we made essential modifications to enhance the clarity of certain questions. Additionally, we evaluated the time needed to complete the questionnaire, which varied from 9 to 16 minutes, and achieved an overall response rate of 90.1%.

### 2.6. Ethical considerations and approval

The Ethics Committee of King Khalid University (IRB = ECM#2023-3205) granted ethical clearance for this study, and the research followed the ethical standards outlined in the Declaration of Helsinki of 1964 and its subsequent amendments, or comparable ethical standards. After a thorough explanation of the study objectives, all participants gave their informed consent. We assured participants of the confidentiality of their provided information and guaranteed that no personally identifying details would be utilized in the study. The grant number for this project GRP 164/44.

### 2.7. Statistical analysis

Data were managed and analyzed using Statistical Package for the Social Sciences software version 27.0 (SPSS Inc., Chicago, IL). Numerical variables were described by the mean and standard deviation, whereas nominal and categorical variables were described by the percentage (%). Distributions of sex and lifestyle factors were analyzed by Pearson *χ*^2^. When the assumptions *χ*^2^ were violated, we used Fisher exact or Monte Carlo test. The Student *t* test was used to compare anxiety and depression levels between groups. The binary logistic regression model was used to identify the predictors of IBS, including different socioeconomic factors, depression, anxiety, and well-being. Odds ratios (OR) and their corresponding 95% confidence intervals (CI) were calculated to assess the strength and precision of the associations. A *P* < .05 was considered statistically significant.

## 3. Results

A total of 379 students were included in this study. The sex distribution shows a relatively balanced representation, with 46.7% of the females and 53.3% of the males. Most participants (94.7%) were single and 52.2% were from King Khaled University. The distribution of majors was almost evenly split between health and non-health specialties, with health specialties accounting for 53.3% and non-health specialties for 46.7%. Most of the participants reside in the city (86.0%), while a smaller percentage live in the village (14.0%). Most participants were Saudi (98.4%), with a small percentage being non-Saudi (1.6%) (Table 1).

**Table 1:** Demographic profile of study participants.

Studied variables (N = 379)		Frequency	Percent
Age	Mean ± sd	21.7 ± 2.9	
Sex	Female	177	46.7
	Male	202	53.3
Marital status	Single	359	94.7
	Married/divorced	20	5.3
Monthly income	<5000 Rial	78	20.6
	5000–10000 Rial	102	26.9
	15,000–20,000 Rial	109	28.8
	More than 20,000 Rial	90	23.7
University	King Saud University Health Sciences	100	26.4
	King Khaled university	198	52.2
	Besha University	81	21.4
Majoring	Health Specialties	202	53.3
	Administrative specialities	55	14.5
	Scientific disciplines	51	13.5
	Literary disciplines	39	10.3
	Engineering majors	30	7.9
	Scientific	2	0.5
Living	City	326	86.0
	Village	53	14.0
Nationality	Non-Saudi	6	1.6
	Saudi	373	98.4

IUSD = 3.75 Riyal.

Approximately three-fourths (72.8%) of participants had not been diagnosed with IBS. About 31.9% of participants did meet the Rome Criteria IV for an IBS diagnosis and 47.2% reported a diagnosis of IBS among a first-degree family member. Almost three-fifths of the participants (57.3%) reported getting 6 to 8 hours of sleep per night and 47.8% of participants did not engage in regular exercise, and 80.7% were nonsmokers, while a smaller percentage either currently smoke or have quit within different time frames (Table 2).

**Table 2:** Health and lifestyle characteristics of study participants with a focus on irritable bowel syndrome and related factors.

Health and lifestyle characteristics	Level	n	%
Have you been diagnosed with irritable bowel disease?	No	276	72.8
	Yes	103	27.2
Having IBS based on Rome criteria IV	No	258	68.1
	Yes	121	31.9
Has irritable bowel syndrome been diagnosed among a first-degree family member?	No	200	52.8
	Yes	179	47.2
Sleeping hours	Less 6 h	93	24.5
	6 to 8 h	217	57.3
	More than 8 h	69	18.2
Exercise 20 min a day	Do not do exercise	181	47.8
	Less 3 weekly	119	31.4
	3 to 5 weekly	59	15.6
	More than 5 weekly	20	5.3
Smoking	Smoke (cigarettes, e-cigarettes, hookah)	58	15.3
	I stopped smoking <6 mo ago	5	1.3
	I stopped smoking more than 6 mo ago	10	2.6
	Nonsmoker	306	80.7

There was a significant association between marital status and IBS. Participants who were married or divorced showed a significantly lower prevalence of IBS compared to those who were single (52.6% vs 30.8%, *χ*^2^ = 3.95, *P* = .047). The highest prevalence of IBS was observed among students of literary and scientific disciplines (*χ*^2^ = 0.952, *P* = .049). There was no significant association found between sex, monthly income, smoking status, living location, sleeping hours, physical activity, and IBS (Table 3).

**Table 3:** Association between sociodemographic variables and the presence of irritable bowel syndrome (IBS) among study participants.

Variables		Having IBS				*χ* ^2^	*P*
		No (n = 258)		Yes (n = 121)			
Sex	Female	116	65.5	61	34.5	0.98	.321
	Male	142	70.3	60	29.7		
Marital status	Married/divorced	249	69.2	111	30.8	4.0	.074
	Single	9	47.4	10	52.6		
Monthly income	<5000 Rial	52	66.7	26	33.3		
	5000 to 10,000 Rial	70	68.6	32	31.4	0.12	.99
	15,000 to 20,000 Rial	75	68.8	34	31.2		
	More than 20,000 Rial	61	67.8	29	32.2		
University	Besha University	45	55.5	36	44.2	6.76	.034
	King Khaled University	128	64.6	70	35.4		
	King Saud University Health Sciences	74	74.0	26.0	24.5		
Majoring	Administrative specialities	41	74.5	14	25.5	0.95	.049
	Engineering majors	19	63.3	11	36.7		
	Health specialties	146	72.3	56	27.7		
	Literary disciplines	20	51.3	19	48.7		
	Scientific disciplines	32	60.4	21	39.6		
Smoking	I stopped smoking <6 mo ago	3	60.0	2	40.0	1.84	.606
	I stopped smoking more than 6 mo ago	5	50.0	5	50.0		
	Nonsmoker	209	68.3	97	31.7		
	Smoke (cigarettes, e-cigarettes, hookah)	41	70.7	17	29.3		
Living	City	224	68.7	102	31.3	0.44	.509
	Village	34	64.2	19	35.8		

*χ*^2^: Chi-square test.

**Figure 1.**
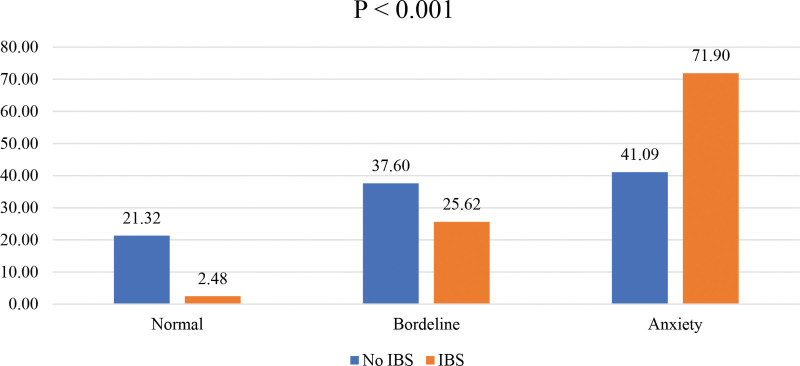
Prevalence of anxiety among students with and without irritable bowel syndrome.

Individuals with IBS demonstrate a significantly higher prevalence of anxiety (71.90%) compared to those without IBS (41.09%). Individuals without anxiety or having borderline anxiety were more prevalent among those without IBS (21.32%, 37.60%), respectively, compared to those with IBS. The difference between both groups was statistically significant (*P* < .001) (Fig. 1).

**Figure 2.**
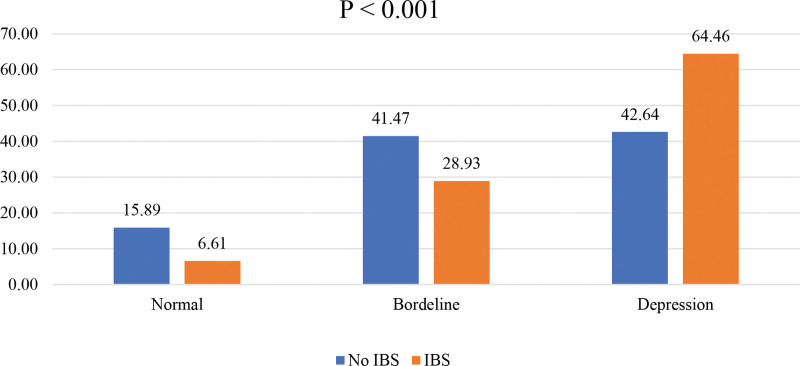
Prevalence of depression among students with and without irritable bowel syndrome.

Students without IBS showed a higher prevalence of normal score of depression (15.89%) compared to those with IBS (6.61%). The prevalence of borderline depression was higher among students without IBS (41.47%) compared to those with IBS (28.93%). In contrast, students with IBS demonstrated a higher prevalence of depression (64.46%) compared to those without IBS (42.64%). This difference was statistically significant *P* < .001 (Fig. 2).

**Figure 3.**
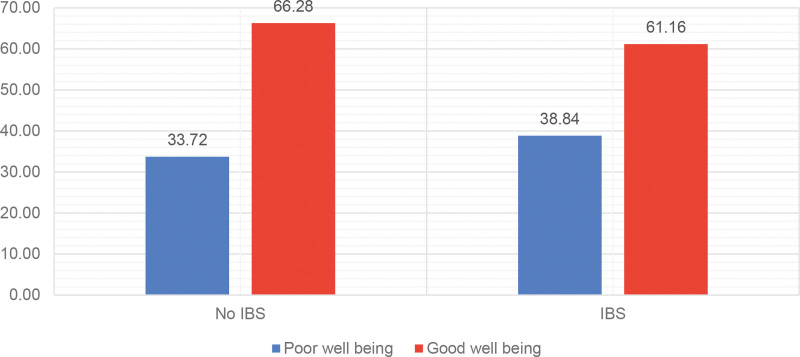
The well-being of students with and without irritable bowel syndrome.

Individuals with IBS had a slightly higher prevalence of poor well-being (38.8%) compared to those without IBS (33.7%). However, this difference was not statistically significant (Fig. 3).

In multivariate analysis, having a family history of IBS [OR = 1.75 (95% CI, 1.06–2.87), *P* = .029] having borderline anxiety [OR = 7.58, 95% CI (2.12–27.06), *P* = .012], having anxiety [OR = 16.07, 95% CI (4.57–56.52), *P* < .001], and having depression [OR = 2.97, 95% CI (1.13–7.83), *P* = .010] were the main significant predictors of IBS among university students (Table 4).

**Table 4:** Predictors of irritable bowel syndrome among the studied university students including depression, anxiety, and well-being.

Variable	S.E.	Wald	*P*	OR	95% OR	
					Lower	Upper
Constant	1.54	1.87	.172	0.12		
Ag	0.04	3.08	.079	1.08	0.99	1.18
Sex (male)	0.28	0.35	.552	0.85	0.50	1.46
Marital status (single)	0.59	1.69	.193	0.47	0.15	1.47
Monthly income		3.07	.381			
Monthly income (<5000 SAR)	0.36	0.01	.914	1.04	0.52	2.09
Monthly income (5000–10,000 SAR)	0.36	1.94	.164	0.60	0.30	1.23
Monthly income (10,001–15,000)	0.38	0.05	.820	0.92	0.43	1.94
University (King Khalid)	0.26	0.01	.929	1.02	0.61	1.71
Majoring (Health)	0.25	2.62	.106	1.51	0.92	2.48
Smoking (current smoker)	0.36	1.81	.179	1.62	0.80	3.26
Living (Urban)	0.37	1.05	.307	0.69	0.33	1.42
Nationality (Saudi)	1.03	0.10	.754	1.38	0.19	10.33
Family member IBS (yes)	0.25	4.78	**.029**	1.75	1.06	2.87
Sleeping hours		1.91	.386			
Sleeping hours (6–8 h)	0.35	1.87	.171	1.62	0.81	3.21
Sleeping hours (more than 8 h)	0.39	0.62	.433	1.36	0.63	2.92
Exercise 20 minutes per day		6.22	.102			
Exercise 20 minutes per day (<3 times/wk)	0.66	0.38	.539	1.50	0.41	5.39
Exercise 20 minutes per day (3–5 times/wk)	0.70	0.39	.530	1.55	0.39	6.13
Exercise 20 minutes per day (>5 times/wk)	0.67	2.45	.118	2.83	0.77	10.46
Anxiety status		21.50	**.0001**			
Anxiety (borderline)	0.30	6.34	**.012**	7.58	2.12	27.06
Anxiety	0.64	18.74	**.0001**	16.07	4.57	56.52
Depression status		8.98	**.011**			
Depression (borderline)	0.49	4.88	.472	1.427	0.54	3.76
Depression	0.29	6.62	**.010**	2.97	1.13	7.83
Well-being (good)	0.27	0.20	.658	0.89	0.52	1.52

Bold values are significant level.

Binary logistic regression model, the dependent variable is being diagnosed with IBS, *R*^2^ = 26%, correct classification is 71%, model is significant *P* < .01, Hosmer and Lemeshow test *P* > .05. OR: Odds ratio; S, E: Standard error. SAR: Saudi Riyal (1 UDS = 3.75 Saudi Riyal). Reference categories are female, being married, having an income of 15,000-20,000 SAR, sleeping hours <6 h, nonpracticing exercise, having no anxiety, and having depression.

## 4. Discussion

### 4.1. The main study findings

The main objective of this study was to assess the prevalence of IBS among university students (health or non-health specialties) using the Rome IV criteria for diagnosis. Additionally, the study aimed to explore associated factors, including sociodemographic characteristics and psychological factors (depression, anxiety, and overall well-being) which could be associated with the prevalence of IBS within this population. In this study, nearly one-third of the participants had IBS. The bivariate analysis revealed that individuals who were unmarried and students in literary and scientific disciplines exhibited the highest prevalence of IBS. Notably, individuals with IBS displayed a significantly higher prevalence of anxiety and depression compared to their counterparts without IBS. Anxiety, borderline anxiety, depression, and a family history of IBS were identified as significant predictors of IBS in multivariate analysis.

### 4.2. Interpretation of the main study findings

There is a variation in IBS prevalence across different countries that may indeed be attributed to the diagnostic tools utilized. In the current study, nearly one-third of the participants had IBS with higher prevalence among non-health students. Das et al^[21]^ reported a higher prevalence of IBS when they used Rome III criteria to diagnose IBS among students from Bengali universities. On the other hand, a lower prevalence rate of approximately 20.0% was observed among Lebanese university students when the Rome III criteria were used for diagnosing IBS.^[22]^ Yang et al^[23]^ found that the pooled prevalence of IBS among Chinese university students in 22 studies was 11.89% (95% CI = 8.06%, 16.35%). Specifically, prevalence rates were 10.50% (95% CI = 6.80%, 15.87%) according to the Rome II criteria, 12.00% (95% CI = 8.23%, 17.17%) according to the Rome III criteria, and 3.66% (95% CI = 2.01%, 6.60%) according to the Rome IV criteria.

Our initial findings suggested that non-health university students had a higher prevalence of IBS compared to health students. However, when performing a multivariate analysis, we did not find a confirmed association between the study field and prevalence of IBS. Indeed, Mohammed et al^[23]^ reported a higher prevalence of IBS among medical students compared to nonmedical students, with rates of 34.4% versus 17.2%, respectively. This finding was also supported by Wani et al,^[24]^ who similarly observed a significantly higher prevalence of IBS among medical students.

Although the exact cause of IBS is not fully understood, it is believed to involve a complex interaction between biological, psychological, and social factors. Psychosocial factors have been found to significantly influence the onset, severity, and course of IBS symptoms.^[25]^ The association between IBS and psychosocial factors has been extensively studied.^[21,26,27]^ Studies have also revealed the indirect mediating role of anxiety in the association between IBS and brain volumes, shedding light on the functional mechanisms of IBS and its related psychosocial factors.^[28]^ Research aimed to determine the relationship between psychological characteristics and different subtypes and the severity of IBS, highlighted the importance of understanding the psychological aspects of the condition.^[29]^

In the current study, there was a significant association between anxiety and IBS in bivariate and multivariate analysis. In fact, stress and anxiety are known to exacerbate IBS symptoms. We found that having IBS increases the odds of having IBS by 16 times. In the same line, Abdelaziz et al,^[26]^ reported that 1 unit increase in a patient’s age and Beck Anxiety Inventory score was associated with 68% and 75% greater odds, respectively, for increased IBS symptoms. In addition, many people with IBS report a worsening of symptoms during periods of increased stress or anxiety.^[29]^ Stress can activate the hypothalamic–pituitary–adrenal (HPA) axis, leading to alterations in gut motility, sensitivity, and immune function, which can contribute to IBS symptoms.^[30]^ Additionally, there is a higher prevalence of psychological disorders, such as depression and anxiety, among individuals with IBS. Psychological distress can exacerbate the perception of abdominal pain and discomfort in people with IBS.^[31]^ Additionally, the experience of living with a chronic condition like IBS can itself lead to psychological distress.^[32]^

According to a clinic-based study, the prevalence of depression among IBS patients is 37.1%.^[27]^ We observed a significant association between depression and IBS in both bivariate and multivariate analysis. Specifically, individuals with depression had a threefold increased probability of having IBS compared to those without depression. This association between depression and IBS has been addressed in numerous studies, indicating the potential importance of psychological factors in the development and exacerbation of IBS symptoms.^[33–35]^ Eijsbouts et al,^[36]^ identified and confirmed 6 genetic susceptibility loci for IBS. These loci included NCAM1, CADM2, PHF2/FAM120A, DOCK9, CKAP2/TPTE2P3, and BAG6. Notably, the first 4 genes are associated with mood and anxiety disorders, expressed in the nervous system, or both. However, coping strategies and behavioral responses to stress and depression can also impact IBS symptoms. Some individuals may engage in maladaptive coping mechanisms, such as avoiding social situations or specific foods, which can further restrict their lives and worsen symptoms.^[37]^ Conversely, effective coping strategies, such as stress management techniques, relaxation exercises, and cognitive behavioral therapy, can help manage and reduce IBS symptoms.^[38]^

It is important to note that while psychosocial factors play a significant role in IBS, they do not imply that IBS is a purely psychological condition. IBS is considered a multifactorial disorder with a complex interplay between biological and psychosocial factors. Treatment approaches for IBS often involve addressing both the physiological and psychosocial aspects of the condition to improve symptom management and overall well-being. That is emphasizing the need for a comprehensive understanding of the interplay between psychological factors and the pathophysiology of IBS.^[39]^

The family history of IBS was associated with increasing the odds of having IBS by a factor of 1.75. Similarly, Saito et al^[40]^ reported that IBS exhibits strong familial aggregation, with variations by relative relationship, suggesting potential genetic or shared environmental etiologies. Relatives of an individual with IBS are 2 to 3 times as likely to have IBS themselves. These findings underscore the importance of genetic and environmental factors in the development and manifestation of IBS.

### 4.3. Strength and limitations

The study addressed different risk factors, including sociodemographic characteristics and lifestyle habits that may contribute to the development of IBS. Furthermore, it investigated the relationship between IBS and emotional well-being, anxiety, and depression among university students by using a validated questionnaire. However, we acknowledge that the study lacked strict exclusion criteria, that is, we did not include all the questions typically associated with the Rome V criteria. Another limitation is that we collected data using a self-administrated questionnaire which might lead to some sort of bias. Also, a cross-sectional design limited our findings to association instead of causation.

## 5. Conclusions

The prevalence of IBS among university students is notably high, especially among those in non-health specialties. Additionally, IBS patients tend to exhibit higher rates of depression and anxiety, although the lower rates of well-being observed among students with IBS were not statistically significant. The main determinants identified for having IBS among students included a family history of IBS, depression, and anxiety. The high prevalence of IBS among university students requires increased awareness, multidisciplinary support, and accessible mental health services. Educational campaigns, collaborations, stress management programs, research, and improved healthcare accessibility are crucial for student well-being.

## Author contributions

**Conceptualization:** Ayoub Ali Alshaikh, Syed Esam Mahmood, Fatima Riaz, Mohammed Abdullah M. AlJebreel, Ramy Mohamed Ghazy.

**Data curation:** Ayoub Ali Alshaikh, Fatima Riaz, Mohammed Abdullah M. Shlwan, Faisal Naser A. Almuidh, Ramy Mohamed Ghazy.

**Formal analysis:** Ayoub Ali Alshaikh, Omar Aodah S. Alshahrani, Ramy Mohamed Ghazy.

**Funding acquisition:** Sultan Mana Alamri, Syed Esam Mahmood, Abdulrahman Saeed H. Almuaddi.

**Investigation:** Abdulrahman Saeed H. Almuaddi, Mohammed Abdullah M. AlJebreel, Ramy Mohamed Ghazy.

**Methodology:** Sultan Mana Alamri, Mohammed Abdullah M. Shlwan, Mohammed Alhussain M. Asiri, Ramy Mohamed Ghazy.

**Project administration:** Ramy Mohamed Ghazy.

**Software:** Nawaf Yahya Y. Al Qasim.

**Supervision:** Ramy Mohamed Ghazy.

**Validation:** Ramy Mohamed Ghazy.

**Visualization:** Ramy Mohamed Ghazy.

**Writing – original draft:** Ramy Mohamed Ghazy.

**Writing – review & editing:** Ramy Mohamed Ghazy.
